# Hyaluronan-based extracellular matrix under conditions of homeostatic plasticity

**DOI:** 10.1098/rstb.2013.0606

**Published:** 2014-10-19

**Authors:** Juan Carlos Valenzuela, Christopher Heise, Gilbert Franken, Jeet Singh, Barbara Schweitzer, Constanze I. Seidenbecher, Renato Frischknecht

**Affiliations:** 1 Department for Neurochemistry and Molecular Biology, Leibniz Institute for Neurobiology Magdeburg, Magdeburg, Germany; 2 Center for Behavioral Brain Sciences (CBBS) Magdeburg, Magdeburg, Germany

**Keywords:** MMP, synaptic plasticity, perineuronal nets, brevican, ADAMTS4, extracellular proteolysis

## Abstract

Neuronal networks are balanced by mechanisms of homeostatic plasticity, which adjusts synaptic strength via molecular and morphological changes in the pre- and post-synapse. Here, we wondered whether the hyaluronic acid-based extracellular matrix (ECM) of the brain is involved in mechanisms of homeostatic plasticity. We hypothesized that the ECM, being rich in chondroitin sulfate proteoglycans such as brevican, which are suggested to stabilize synapses by their inhibitory effect on structural plasticity, must be remodelled to allow for structural and molecular changes during conditions of homeostatic plasticity. We found a high abundance of cleaved brevican fragments throughout the hippocampus and cortex and in neuronal cultures, with the strongest labelling in perineuronal nets on parvalbumin-positive interneurons. Using an antibody specific for a brevican fragment cleaved by the matrix metalloprotease ADAMTS4, we identified the enzyme as the main brevican-processing protease. Interestingly, we found ADAMTS4 largely associated with synapses. After inducing homeostatic plasticity in neuronal cell cultures by prolonged network inactivation, we found increased brevican processing at inhibitory as well as excitatory synapses, which is in line with the ADAMTS4 subcellular localization. Thus, the ECM is remodelled in conditions of homeostatic plasticity, which may liberate synapses to allow for a higher degree of structural plasticity.

## 1. Introduction

Neuronal networks maintain stable function despite multiple plastic challenges. Destabilizing influences such as long-lasting deflection or attenuation of excitation can be counterbalanced by mechanisms of homeostatic plasticity. This form of plasticity preserves network stability, adjusting synaptic strength within the network by profound molecular changes within the pre- and the post-synapse [1–3]. From the extracellular side the brain's extracellular matrix (ECM) influences various forms of synaptic plasticity. The brain's ECM enwraps cell bodies and proximal dendrites and interdigitates with synaptic contacts [4]. It is found on virtually all neurons in the mature brain; however, its most prominent form is the so-called perineuronal net (PNN) that has a mesh-like appearance and is mainly, but not exclusively, found on parvalbumin (PV)-positive interneurons [5]. On the molecular level, the ECM is made of the polymeric carbohydrate hyaluronic acid (HA) that forms the backbone of a meshwork consisting of proteoglycans belonging mainly to the lectican family of chondroitin sulfate proteoglycans (CSPGs) [6,7]. This family comprises four abundant components of brain ECM of which aggrecan and brevican, a lectican that is primarily contributed by glial cells, are most prominent in the mature nervous system.

It was hypothesized that the appearance of the mature ECM is functionally involved in the switch from developmental to adult modes of synaptic plasticity [4]. This is supported by the finding that CSPG-containing ECM acts to inhibit neurite outgrowth and regeneration [8]. Further, experimental removal of the ECM induces increased spine motility and activity-dependent neuronal network rearrangements in rat visual cortex, and thus reinstates a mode of plasticity characteristic of the juvenile animal [9,10]. Moreover, we have recently reported that ECM removal not only enhances network rearrangements in response to sensory stimuli, but also promotes cognitive flexibility in rodents [11]. Thus, in the mature brain, HA-based ECM stabilizes established neuronal networks at the cost of plasticity. However, there is still limited activity-dependent structural plasticity observed in the adult brain in the presence of ECM [12]. Therefore, we assume there are intrinsic molecular mechanisms modulating the ECM to remove or convert non-permissive cues into permissive ones. In this context, several proteases have been reported to promote activity-dependent ECM degradation and structural plasticity [13,14]. Importantly, also CSPGs such as brevican and aggrecan are proteolytically processed by a large number of proteases, mainly from the matrix metalloprotease (MMP) family [15,16]. The lecticans, and especially brevican, are most efficiently cleaved by a disintegrin and metalloproteinase with thrombospondin motifs 4 (ADAMTS4) at a specific site [15–17]. ADAMTS4 cleavage separates the N-terminal HA-binding globular domains (G1) from the C-terminal globular domains (G3) of brevican and thus may disintegrate ECM structure [18]. However, whether brevican and thus ECM processing by ADAMTS4 are controlled by neuronal activity and therefore may play an important role in synaptic plasticity remains unknown. Here, we analysed brevican processing in rat brain slices and in neuronal cultures and tested whether brevican processing is altered during network inactivation, which leads to homeostatic changes of synaptic strength and to structural plasticity.

## 2. Results

### (a) Detection of proteolytic fragments of brevican

**Figure 1.**
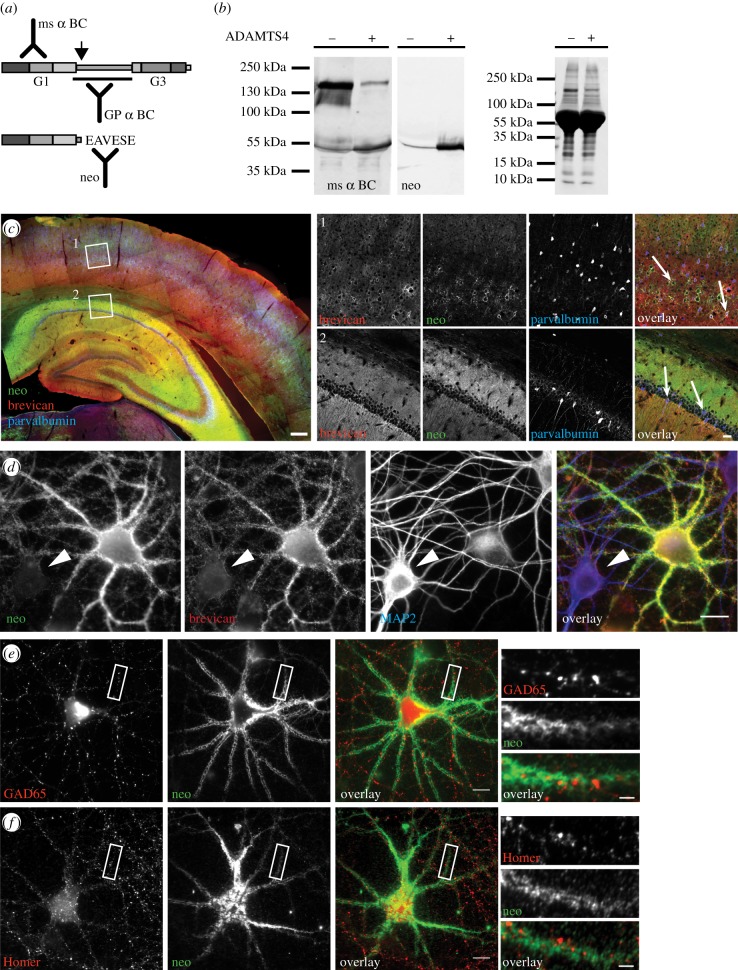
The N-terminal proteolytic fragment of brevican is found in PNNs and peri-synaptically. (*a*) Schematic of brevican with its N- and C-terminal globular domains G1 and G3 and location of antibody epitope regions (ms, mouse; GP, guinea pig). Arrow indicates the ADAMTS4 cleavage site yielding the proteolytic fragment (below). Neo antibody is produced against the newly emerging C-terminus after processing by ADAMTS4. (*b*) Western blots from supernatants of HEK293T cells overexpressing brevican with (+) or without (−) ADAMTS4 co-expression. Blots probed with the anti-G1 domain antibody (left; ms α BC) show the full-length band at 145 kDa and the proteolytic N-terminal fragment at 53 kDa. Note the increase of the 53 kDa band after co-expression with ADAMTS4. Middle: same samples probed with the neo antibody. Only the ADAMTS4-specific proteolytic band at 53 kDa is detected. Right: Coomassie blue staining of loaded samples. Note that the major band at 55 kDa represents serum albumin present in the cell culture media. (*c*) Brain slice from adult rat brain stained with gp α brevican (red), neo antibody (green) and anti-parvalbumin (blue). There is a large overlap between gp α brevican and neo staining. Note the strong staining of the molecular layer with the neo antibody while in the cortex mainly a cellular staining is observed. Boxed areas are represented at higher magnification (right). (i) Note that the cellular staining is to a large extent found on parvalbumin-positive interneurons in the cortex (arrows in 1). Also in the hippocampus cellular staining is found on parvalbumin-positive interneurons (arrows in 2). However, in addition a strong staining of the molecular layer is observed. (Scale bars, 100 μm, inset: 20 μm.) (*d*) Staining of dissociated cortical cultures at DIV 24 using neo (green) and gp α brevican (red) antibody co-stained with the dendritic marker MAP2 (blue). In addition to the very dominant staining on a subset of neurons virtually all cells displayed immunoreactivity with gp α brevican and to a lesser extent also with neo antibody (arrowhead). (*e*) Neo antibody staining (green) is found around inhibitory (GAD65; red) and (*f*) excitatory synapses (Homer; red). (Scale bars, 10 μm, insets: 2 μm.)

Brevican can be processed by a large number of extracellular proteases [15]. *In vitro* studies showed that one of the most efficient brevican-cleaving enzymes is ADAMTS4 [15]. The cleavage site in the central region (after Glu395) shows high similarity to aggrecan and other lecticans [15,18,19]. So far, the regulatory mechanisms of brevican cleavage as well as those of ADAMTS4 activation are not known. In order to monitor and quantify proteolytic cleavage of brevican by ADAMTS4 under conditions of homeostatic plasticity, we raised antibodies against the newly exposed amino acids at the C-terminus of the N-terminal fragment after ADAMTS4 cleavage (neo epitope, figure 1*a*), which have previously been reported to detect exclusively the N-terminal cleavage product of brevican but not the full-length protein [15,19,20]. First, we tested neo antibody specificity on Western blots of cell culture supernatants from human embryonic kidney (HEK) 293T cells overexpressing brevican alone or brevican together with ADAMTS4 (figure 1*b*). The 53 kDa N-terminal cleavage product was present in the supernatant of ADAMTS4/brevican co-transfected cells, but barely detectable in brevican-only-expressing cells (figure 1*b*). Immunoreactivity with the neo antibody proved that the 53 kDa N-terminal fragment was derived from ADAMTS4 cleavage. It is of note that this antibody did not detect the full-length protein (figure 1*b*). Thus, the neo antibody is a valuable tool to detect exclusively the ADAMTS4-derived cleavage fragment of brevican and thus to monitor ADAMTS4 activity.

### (b) Distribution of ADAMTS4-derived brevican fragments in brain

To check for effects of cleavage on membrane association of brevican, we analysed the distribution of proteolytic fragments in subcellular fractions from rat brain (electronic supplementary material, figure S1). As expected, we detected full-length brevican in all brain fractions, with the strongest intensity in fraction S2 where soluble components of the ECM are found [21] (electronic supplementary material, figure S1*a*,*b*). In accordance with previous reports, brevican was also found in the myelin (My), synaptosome (Syn) and light membrane (LM) fractions, although to a much lesser extent [22]. In addition, the 53 and 80 kDa proteolytic fragments were not only most prominent in the S2 fraction, but also present in membrane-containing fractions such as synaptosomes, light membranes or myelin (electronic supplementary material, figure S1*a*,*c*). However, when we compared the relative abundance of full-length brevican and its C-terminal 80 kDa fragment across the fractions (ratio 80/145 kDa band), we found the fragment enriched in the soluble fractions S1 and S2 (electronic supplementary material, figure S1*a*), which is not the case for the 53 kDa portion (electronic supplementary material, figure S1*b*). Taken together, this suggests a stronger association of the HA-binding N-terminal fragment to neural membranes compared with full-length or 80 kDa brevican.

### (c) Brevican fragment is enriched on parvalbumin-positive interneurons

To study the spatial distribution of brevican processing in mature rodent brain, we investigated the localization of the ADAMTS4-derived proteolytic fragment in relation to the distribution of total brevican in rat brain sections by immunohistochemistry. While brevican staining was observed as diffuse labelling in most brain regions including hippocampus and cortex, in addition it appeared as a dense net-like structure typical for PNNs around a subset of cells, which were mostly parvalbumin-positive interneurons (figure 1*c* and electronic supplementary material, figure S2*a*–*d*). Staining with the neo antibody appeared very similar to total brevican staining with some deviations. In the molecular layer of the hippocampus, the neo antibody immunoreactivity mainly had a diffuse appearance (figure 1*c*). This may represent labelling of axonal coats at perisynaptic sites as recently described in human tissue [23]. However, in cortical regions, neo epitope labelling showed mainly a PNN-like staining pattern, especially in layer IV, as described previously [24]. Thus, brevican cleavage is an abundant phenomenon in most brain regions where it may occur at synapses and within PNNs.

### (d) Brevican is processed in dissociated cortical cultures

**Figure 2.**
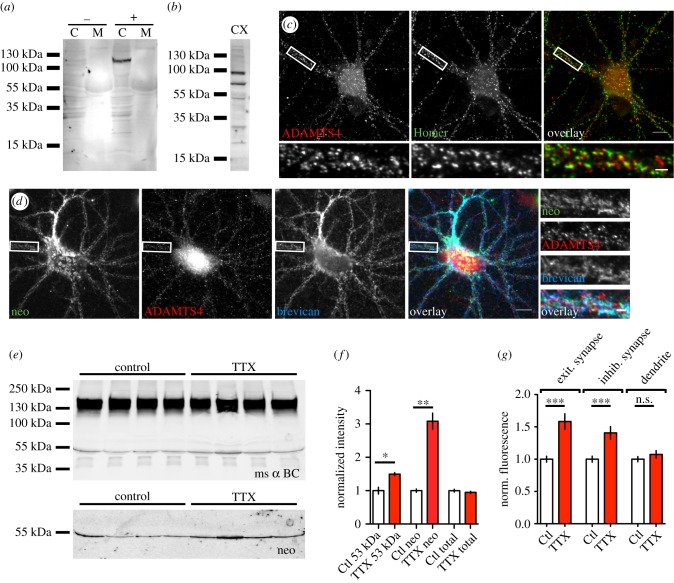
Brevican is proteolytically processed at synapses after prolonged network inactivation. (*a*) Western blots of cell lysates (C) and medium (M) of HEK293T cells transfected with ADAMTS4-EGFP (+) and control cells (−). An antibody against the proteolytic domain of ADAMTS4 specifically detects the fusion protein at 130 kDa in the cell lysate. (*b*) Western blot of cell lysate from high-density cortical cultures at DIV24 probed with anti-ADAMTS4 antibody. Note the bands at about 100, 75 and 50 kDa representing full-length, active and C-terminally truncated versions of ADAMTS4, respectively. (*c*) Co-staining of ADAMTS4 (red) and the excitatory synapse marker Homer (green) in cortical primary cultures at DIV24. Note the large degree of overlap of the two stainings. (*d*) Co-staining of neo antibody (green) with ADAMTS4 (red) and gp α brevican (blue). Boxed areas are shown as high magnification to the right. (Scale bars, 10 μm, insets: 2 μm.) (*e*) Western blot of supernatants from dissociated cortical cultures at DIV24 supplied with excess of full-length brevican detected with anti-G1 antibody (ms α BC; upper blot) and with the neo antibody (lower blot). Detected bands at 145 and 53 kDa represent the full-length and the N-terminal proteolytic fragment of brevican, respectively. Treatment with TTX leads to a marked increase in the intensity of the 53 kDa band. (*f*) Quantification of immunoreactive bands as depicted in (*d*). There is a strong increase in the 53 kDa band after TTX treatment for 48 h (G1 antibody: Ctl = 1.00 ± 0.10, TTX = 1.50 ± 0.04; *p* = 0.001; neo: Ctl = 1.00 ± 0.05, TTX = 3.08 ± 0.24; *p* = 0.001, total brevican: Ctl = 1.00 ± 0.04, TTX = 0.95 ± 0.03; *p* = 0.372; *n* = 4). Note that there is an equal amount of total brevican loaded in all lanes. (*g*) Quantification of neo staining in dissociated cortical cultures at Homer-positive excitatory synapses, GAD65-positive inhibitory synapses and on MAP2-positive dendrites. There was an increase of brevican cleavage at excitatory (Ctl = 1 ± 0.05, TTX = 1, 58 ± 0.12; *n* = 72; *p* < 0.0001) and inhibitory synapses (Ctl = 1 ± 0.05, TTX = 1, 41 ± 0.10; *n* = 64; *P* = 0.0003) but not on dendrites (Ctl = 1 ± 0.04, TTX = 1, 07 ± 0.06; *n* = 62; *p* = 0.309) detected (mean ± s.e.m., unpaired Student's *t*-test).

Others and we reported previously that PNN-like structures develop *in vitro* in dissociated neuronal cultures, making them an excellent model by which to study ECM-linked molecular events during synaptic plasticity [25–27]. Therefore, we analysed the localization of the ADAMTS4-derived fragment of brevican in cortical cultures at DIV (days *in vitro*) 21–24, a time point when the mature form of the ECM is fully developed in these cultures [27]. We found brevican staining on virtually all neurons, albeit with different staining qualities (figures 1*d* and electronic supplementary material, figure S1*d*). The largest population of neurons exhibited a moderate staining and represented most likely MAP2-rich excitatory neurons (figure 1*d*, arrowhead). However, the staining on a subpopulation of cells was more intense and had a net-like appearance as observed in brain slices (figure 1*d* and electronic supplementary material, figure S2*a*–*d*). Indeed, cells bearing PNN-like structures were co-stained with the inhibitory cell marker anti-GAD65 and partially also with PV (electronic supplementary material, figure S2*b*,*c*). Neo epitope labelling exhibited a weak, dotted pattern on most neurons (figures 1*d* and electronic supplementary material, figure S2*a*–*d*). However, on a small number of GAD65-positive interneurons, the staining was intense and had a typical PNN-like appearance. The staining co-localized well with pan brevican antibody (figures 1*d*,2*d* and electronic supplementary material, figure S2*b*–*d*). High-resolution images reveal the net-like appearance of the neo epitope surrounding inhibitory as well as excitatory synapses (figure 1*e*,*f*). Thus, the ADAMTS4-derived N-terminal fragment of brevican exhibits a similar distribution in dissociated cultures as *in vivo*, and is found associated with PNNs as well as peri-synaptically.

### (e) ADAMTS4 is expressed in dissociated cultures and associates with synapses

The presence of the brevican fragment immunoreactivity in culture points to expression of active ADAMTS4 under culture conditions. To prove this, we analysed ADAMTS4 immunoreactivity with an antibody directed against the proteolytic domain of ADAMTS4. This antibody was shown to be specific, because on HEK293T cells overexpressing ADAMTS4 fused to EGFP it detected a band of approximately 125 kDa in the cell lysate, representing the full-length protein, whereas no band was detected in untransfected HEK293T cells (figure 2*a*). Next, we probed Western blots from cells lysates of dissociated high-density cortical cultures (figure 2*b*). It has previously been reported that ADAMTS4 undergoes a series of activation steps through proteolytic cleavage yielding a characteristic pattern of fragments [28,29]. Indeed, we were able to detect clear bands at 100 kDa, representing the endogenous full-length protein. Further, we detected bands of about 70 and 50 kDa, which were previously described as activated and additionally C-terminally truncated versions of ADAMTS4, respectively [30]. Finally, we analysed the subcellular localization of ADAMTS4 in dissociated cortical cultures (figure 2*c*,*d*). ADAMTS4 immunoreactivity was found in a punctate pattern in cell bodies and neurites. Surprisingly, co-staining with the excitatory post-synaptic marker Homer1 revealed that 48 ± 2.1% of all excitatory synapses and 54 ± 2.9% of inhibitory synapses contained ADAMTS4. Thus, ADAMTS4 is indeed expressed and activated in dissociated neuronal cultures and further associates to a large degree with synapses.

### (f) Increased processing of brevican after prolonged network inactivation

The ECM of the brain has been implicated in reducing structural plasticity in the adult. Therefore, we wondered whether treatments, which are known to induce plasticity, would alter processing of brevican as a major constituent of adult brain ECM. It has previously been reported that prolonged network silencing induces structural and functional changes at synapses in dissociated neuronal cultures [31,32]. Therefore, we silenced neuronal networks to induce homeostatic adaptations by application of 2 μM tetrodotoxin (TTX) for 48 h to the cell culture medium of rat cortical cultures [1]. In a first approach, in order to test for general proteolysis of brevican, we added an excess of recombinant full-length brevican produced in HEK293T cells to the neuronal cultures during the silencing period. After 48 h, supernatants of silenced and control cultures were collected and equal amounts subjected to SDS-PAGE and quantitative Western blotting. As figure 2*e* shows, in all samples both the full-length and the N-terminal brevican fragment were present. Quantification revealed that all samples contained the same amount of total brevican, which was determined by summing the proteolytic fragment and the full-length protein (figure 2*f*). However, the amount of 53 kDa fragment was clearly increased after network silencing, suggesting higher proteolytic activity in treated cultures (figure 2*f*). To prove the involvement of ADAMTS4 in this processing of brevican, we used the neo antibody and indeed found a similar increase of the ADAMTS4-derived fragment in the TTX-treated samples, suggesting ADAMTS4 is the major responsible protease (figure 2*f*).

Next, we examined in which cellular compartment proteolysis occurs. For this purpose, dissociated cortical cultures were treated with TTX for 48 h and subsequently stained with neo epitope antibody. We co-stained neurons with the somatodendritic marker protein MAP2 and used this staining as a mask to determine neo epitope fluorescence intensity as a measure of proteolytic cleavage (figure 1*d* and electronic supplementary material, figure S2*a*). To our surprise, neo staining did not differ between the experimental groups, suggesting no overall change in brevican processing (figure 2*g*). Because the ECM enwraps synapses and thereby may contribute to their stabilization, and because we found ADAMTS4 to be present near synapses, we hypothesized that brevican is differentially processed in the immediate vicinity of synapses. To test this, we used Homer and GAD65 antibodies as markers for excitatory or inhibitory synapses, respectively, and measured fluorescence intensity of neo antibody staining in an area 1 μm around inhibitory or excitatory synapses. Interestingly, we found increased neo immunoreactivity around both synapse types after TTX treatment (figure 2*g*). Taken together, our results suggest that long-term inactivation of neuronal networks leads to increased brevican processing at inhibitory as well as excitatory synapses, which may be a prerequisite for synaptic rearrangements under conditions of homeostatic plasticity.

## 3. Discussion

Structural plasticity, which is thought to be the basis of learning and memory formation, is reduced in adult when compared with adolescent animals [12]. Importantly, components of the mature ECM-like CSPGs are considered to inhibit structural changes in the adult brain and thereby to stabilize synaptic contacts and maintain synapse integrity [4,10,23]. Indeed, spine motility is increased in the rat visual cortex after ECM removal and juvenile forms of structural plasticity are reinstated [9,10]. It is therefore plausible that for learning or adaptation, local and controlled removal or modification of the ECM is required to allow for local structural plasticity [4]. As a model for adaptation, prolonged network inactivation has been shown to strengthen synapses and to lead to profound molecular as well as structural changes in the pre- and post-synaptic compartments *in vivo* and *in vitro* [1–3,33]. Here, we found that brevican cleavage at synaptic sites is a hallmark of this plasticity and thus indicates a crucial involvement of the ECM in homeostatic processes.

### (a) ADAMTS4 removes inhibitory cues from the synapse

Subcellular fractionation suggested that emerging proteolytic fragments are differentially associated with cell membranes. While the C-terminal 80 kDa fragment, which contains the CS side chains, is mainly found in the soluble fractions, the 53 kDa fragment is more tightly associated with membranous fractions such as synaptosomes and light membranes (electronic supplementary material, figure S1). This suggests that brevican cleavage may not only loosen the ECM structure by degrading one of its major components, but also locally removes the non-permissive cue for structural plasticity, the chondroitin sulfates. However, brevican is not the main CS-bearing molecule in the ECM. In fact, it has been suggested this may be the closely related lectican aggrecan [34]. ADAMTS4 cleavage of brevican is very likely accompanied by cleavage of aggrecan, the first known substrate of the enzyme, which was therefore termed aggrecanase-1 [15,16]. Thus, it is very suggestive that also other substrates of ADAMTS4, such as the members of the lectican family [17] and importantly aggrecan, are processed during synaptic plasticity, which indeed would remove a substantial part of the negatively charged CS moieties from the neuronal surface. Considering the similar architecture of the proteins [7], it is plausible that ADAMTS4-mediated cleavage of the lecticans separates the HA-binding N-terminal domains from the CS-binding regions, thereby altering the fine structure of the ECM. Because aggrecan is mainly found in PNN-bearing neurons, cleavage of this molecule would very likely reduce PNN density. Thus, local ADAMTS4 activity removes inhibitory cues from cell membranes and synapses and thereby may promote plastic changes during synaptic plasticity.

### (b) Proteolysis of extracellular matrix molecules generates signalling molecules

Recent work on matrix metalloprotease 9 (MMP9) and the serine protease neurotrypsin suggests that ECM proteolysis may expose new epitopes that exert signalling functions important for synaptic plasticity [35]. Proteolytic activity of MMP9, which is necessary for normal long-term potentiation (LTP) [36] leads to activation of β1-containing integrins and subsequently to a mobilization of synaptic NMDA receptors [37]. This suggests that substrates of MMP9 are activated through proteolytic cleavage and subsequently act as integrin ligands. One possible substrate within the ECM is β-dystroglycan, which is proteolytically cleaved by MMP9 in an activity-dependent manner [38]. However, whether β-dystroglycan activates intergrins is as yet unknown, and thus the major signalling molecule remains elusive.

The serine protease neurotrypsin is secreted from the pre-synapse during high synaptic activity [39]. Its only known substrate so far is agrin [13]. Interestingly, slices from neurotrypisn knockouts exhibit a reduced number of post-LTP filopodia which can be rescued by the application of the 22 kDa agrin fragment. Hence, this neurotrypsin-derived fragment endows signalling properties necessary for filopodia formation. With respect to brevican cleavage, this suggests that in addition to the effect on ECM structure, brevican fragments may also have instructive, signalling functions. This is supported by the finding that proteolytic processing of brevican appears to be a significant extracellular event in the remodelling of the dentate gyrus after entorhinal cortex lesion, and may modulate sprouting and synaptogenesis [40]. In addition, the invasiveness of glioma cells correlates with brevican cleavage and the 53 kDa fragment is mainly responsible for this effect [41]. Therefore, it may well be that ADAMTS4 activity and brevican cleavage not only play a passive role by removing negative cues from synaptic sites. It will be a future challenge to investigate whether ADAMTS4-derived brevican fragments may indeed endow signalling properties that induce structural plasticity and to identify the receptors involved.

### (c) Extracellular matrix modulation and functional plasticity

Indeed, the mature form of the ECM is not only a plasticity-restricting structure, but as a series of works have shown it is important to maintain LTP, also a measure for synaptic plasticity [42]. In fact, a number of mice lacking different components of the ECM show deficits in synaptic plasticity. Maintenance of LTP is dramatically impaired in brevican mutants, a phenotype that can be mimicked by application of anti-brevican antibody. Also mutants for tenascin-R, a direct binding partner of brevican in the ECM, exhibit deficits in both LTP and in long-term depression (LTD). Treatment of hippocampal slices with chondroitinase ABC or hyaluronidase (Hyase), which removes CS from CSPGs or digests HA, respectively, leads to a reduction in LTP expression [43,44]. However, not only LTP and LTD are affected by ECM removal, but also synaptic short-term plasticity [27] as Hyase-treated cells exhibited decreased paired-pulse ratio when compared with control cells. As the mechanism responsible, we have identified increased lateral diffusion of AMPA receptors within the post-synaptic membrane, which accounts for the exchange of desensitized receptors for naive ones [27,45]. Thus, endogenous alterations in the perisynaptic ECM resulting from local activity-related proteolysis are very likely to alter synaptic function and to affect various forms of synaptic plasticity. Whether this is due to removal of negative cues or the formation of new signalling molecules remains to be addressed in future experiments.

## 4. Material and methods

### (a) Antibodies

For a complete list of used antibodies and dilutions, see the electronic supplementary material, section Methods.

### (b) Cell cultures

Primary cultures of cortical neurons for WB and ICC were prepared as described previously [2]. All cells were kept in a humidified incubator with 5% CO_2_. Cells were plated in polystyrene six-well plates (300 000 cells per well) for immunoblots and on poly-l-lysine-coated glass coverslips (50 000 cells per coverslip) for immunocytochemistry. Cell cultures of HEK293T cells stably expressing brevican were cultured as described [46]. A description of the preparation of cell lysates and brevican-conditioned media is given in the electronic supplementary material, section Methods.

### (c) Subcellular fractionation

Subcellular fractionation of adult rat brains was performed as described previously [22].

### (d) Western blotting

Find a detailed description of Western blotting in the electronic supplementary material, section Methods.

### (e) Immunocytochemistry and immunohistochemistry

Immunocytochemistry and immunohistochemistry were performed as described [27,47]. Find further description in the electronic supplementary material, section Methods.

### (f) Image acquisition and analysis

Images were acquired with Zeiss Axio imager A2 (Zeiss Microimaging), a CoolSNAP MYOcamera and Visiview software (version 2.1.1). For each set of coverslips (treatments versus control), the same exposure time was taken. All images were analysed using NIH ImageJ 1.47v or OpenView software (written by N. Ziv and co-workers [48]) with appropriate background subtraction and adjusted for presentation. Graphics were performed with Prism v. 5 software (GraphPad Software).

## Supplementary Material

Supplementary materials
