# Supplementary material for: Hyaluronan-based extracellular matrix under conditions of homeostatic plasticity
Source: Philos Trans R Soc Lond B Biol Sci. 2014 Oct 19;369(1654):20130606. doi: 10.1098/rstb.2013.0606 (PMC4173291; doi:10.1098/rstb.2013.0606)
Supplement: Supplementary materials [file rstb20130606supp1.pdf]

Valenzuela, J.C. *et al.* 2014. Hyaluronan-based ECM under conditions of homeostatic plasticity. *Phil. Trans. R. Soc. B*. **369** doi: 10.1098/rstb.2013.0606

Supplementary materials

Antibodies

The following primary antibodies were used for western blot (WB) and immunocytochemistry (ICC): goat anti-human ADAMTS4 (WB, 1:250, ICC, 1:50, R&D systems, AF4307), mouse anti-brevican (WB, 1:1000, BD Transduction Laboratories), rabbit anti brevican "Neo" (WB and ICC, 1:1000, custom-made; epitope CGGQEAVESE), guinea pig anti-brevican (Seidenbecher et al., 1995) (central region, custom-made), mouse anti-homer1 (ICC, 1:500, SYSY, Cat. 160011), mouse anti-GAD65 (ICC, 1:500, Abcam, Cat. Ab26113), guinea pig anti-MAP2 (ICC, 1:1000, SYSY, Cat. 188004), mouse anti-parvalbumin (ICC, 1:1000, Swant, Cat. 235).

Secondary antibodies used: CF™770-conjugated goat anti-mouse IgG (WB, 1:5000, Biotium, Cat. 20077), Alexa Fluor® 680 donkey anti-goat IgG (WB, 1:5000, Invitrogen, Cat. A21084), Alexa Fluor® 680 goat anti-rabbit IgG (H+L) (WB, 1:5000, Invitrogen, Cat. 21109), Cy™ 3 donkey anti-goat IgG (H+L) (ICC: 1:2000, Dianova, Cat. 705-168-147), Alexa Fluor® 488 donkey anti-mouse IgG (H+L) (ICC, 1:2500, Invitrogen, Cat. A21202), Alexa Fluor® 488 donkey anti-rabbit IgG (H+L) (ICC, 1:2500, Invitrogen, Cat. A21206), Cy™ 5 donkey anti-guinea pig (ICC, 1:2000, Dianova, Cat. 706175148).

Preparation of cell lysates and brevican-conditioned media

Cortical primary neurons were harvested for analysis at 21-28 DIV. Cells were washed briefly two times with PBS buffer and then scraped off and lysed with RIPA buffer (150 mM sodium chloride, Triton X-100, 0.5% sodium deoxycholate, 0.1% SDS, 50 mM Tris, pH 8.0), containing Complete protease inhibitor cocktail (Complete ULTRA tablets, Mini, EDTA-free, EASYpack, Roche). Lysates were cleared by centrifugation at 8,000 x g for 10 min. Next, samples were boiled at 95 °C for 10 min with 4 x loading buffer (40% Glycerol, 240 mM Tris/HCl pH 6.8, 8% SDS, 0.04% bromophenol blue, 5% beta-mercaptoethanol). Conditioned media from HEK 293 cells expressing brevican were collected 72 h after the cells reach 100 % of confluence in the plate. HEK 293T cells transfected with pEGFP-N1-msADAMTS4 and untransfected cells were processed for western blot as described above after 48 h of transfection.

Network inactivation in cortical cultures and brevican cleaveage assay

100 μl of brevican-conditioned media was added to 21DIV cortical neurons containing 200 μl of neurobasal medium (50.000 cells per well, 24 well Polystyrene plate). For network

inactivation cells were treated with 2 µM tetrodotoxin (TTX). After 48 h the media from control and treated cells were collected for western blotting analysis.

Immunocytochemistry

Neurons were fixed in 4% paraformaldehyde in PBS for 5 min at room temperature. Before immunostaining, the samples were permeabilized in 0.2% Triton X-100 in PBS for 5 min and then blocked with blocking solution (10% FCS, 0.1% glycine in PBS) for 45 min at room temperature. Primary antibodies were applied for 2h at room temperature, secondary antibodies for 1 h at room temperature. Both primary and secondary antibodies were diluted in blocking solution. Coverslips were mounted on slides with Mowiol (Calbiochem).

Immunohistochemistry

Adult Wistar rats were anesthetized with isoflurane and subsequently transcardially perfused with PBS followed by fixative containing 4% paraformaldehyde (PFA) in PBS, pH 7.4. The brains were removed from the skull, post-fixed in the same fixative overnight at 4°C, cryoprotected by incubation with 0.5 M and 1 M sucrose, frozen with cold isopentane (precooled at -74°C) and stored at -20°C. Free-floating 30–40 mm thick coronal brain sections were cut using a cryotome, washed with PBS, incubated with 1% Na-borohydride in PBS (to block aldehyde groups from PFA) and washed with PBS again. The slices were then blocked and permeabilized in 10% normal goat serum/0.3% Triton X-100 in PBS for 60 min and incubated overnight at 4°C on a shaker with primary antibodies diluted in the same blocking solution with 0.01% Na-azide. After washing with PBS, brain sections were blocked again with 0.4% BSA/0.3% Triton X-100 in PBS for 60 min followed by overnight incubation with appropriate secondary antibodies diluted in the same blocking solution. Slices were then washed with PBS and mounted onto microscopic glass slides (Menzel, Germany) using DAPI-containing Vectashield (Vector Labs). Images were taken with a Leica SP5 confocal microscope for high resolution images and with a Zeiss Axio Imager A2 (Zeiss Microimaging), a CoolSNAP MYO camera for the overview images.

Western blotting

Samples were subjected to electrophoresis using Tris-glycine SDS polyacrylamide (SDS-PAGE) on 5 –20 % gels under fully reducing conditions and transfer onto PVDF membrane (Roth) was performed according to standard protocols. Western blots were immunodeveloped by overnight incubation at 4°C with primary antiserum goat anti-human ADAMTS4, 2 h with mouse anti-brevican or 2 h with rabbit anti-brevican neo epitope. As secondary antibodies CF™ 770 conjugated goat anti-mouse, Alexa Fluor® 680 donkey anti-goat or Alexa Fluor® 680 goat anti-rabbit were used. Western blot protein detection was performed using a LI-COR Odyssey scanner, which allows simultaneous detection of two

different proteins using secondary antibodies from different species. Protein quantification analysis was performed with NHI ImageJ 1.47v.

Image acquisition and analysis

Images were acquired with Zeiss Axio Imager A2 (Zeiss Microimaging), a CoolSNAP MYO camera, and Visiview software (version 2.1.1). For each set of coverslips (treatments vs control), the same exposure time was taken. All images were analyzed using NIH ImageJ 1.47v or OpenView software (written by N. Ziv; Tsuriel et al., 2006) with appropriate background subtraction and adjusted for presentation. Graphics were performed with Prism 5 software (GraphPad Software).

Subcellular fractionation of brain tissue

For subcelluar fractionation studies adult rat forebrain was homogenised and differentially centrifuged, essentially as described in (Seidenbecher et al., 2002).

Seidenbecher CI, Richter K, Rauch U, Fassler R, Garner CC, Gundelfinger ED (1995) Brevican, a chondroitin sulfate proteoglycan of rat brain, occurs as secreted and cell surface glycosylphosphatidylinositol-anchored isoforms. *J Biol Chem* 270: 27206-27212.

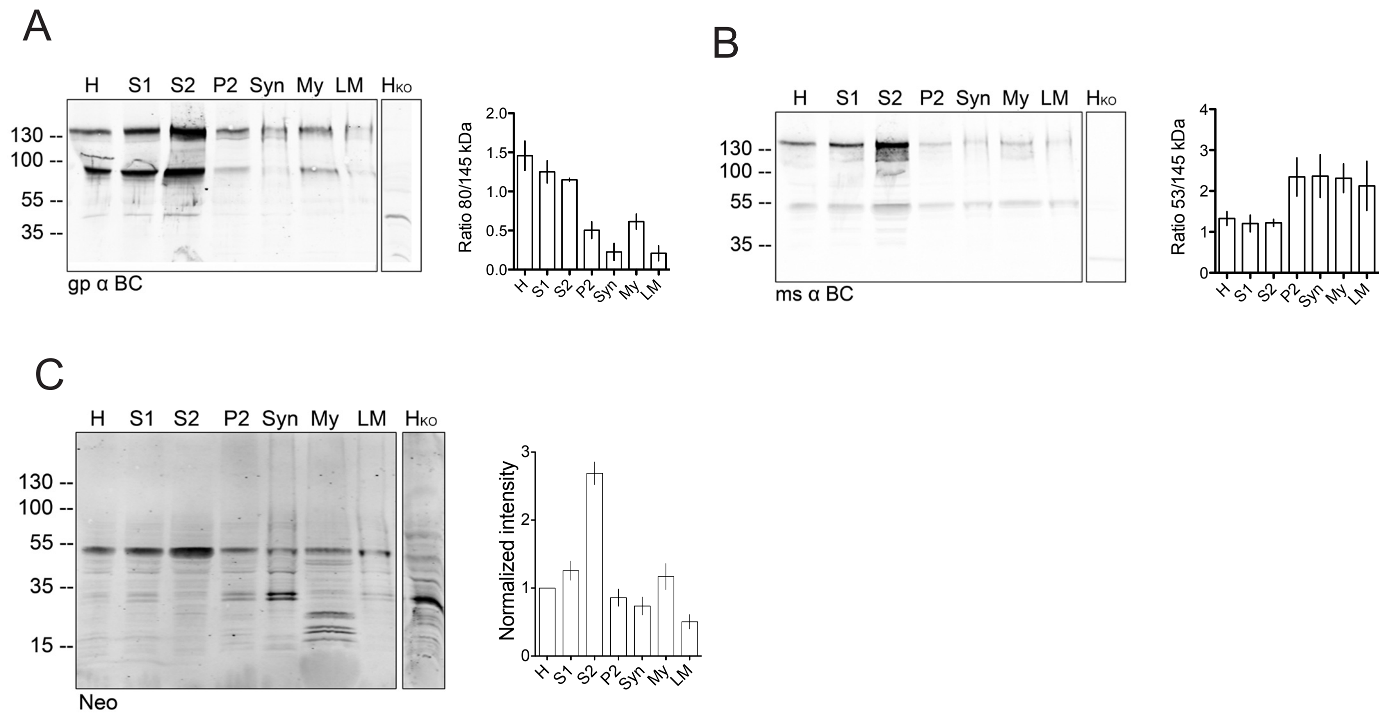

Supplementary Figure 1.

Brevican is differentially associated with neuronal membranes. Western blots were loaded with samples from subcellular fractionations from adult rat brain (20 µg of total protein). A) Western blot probed with anti brevican central domain antibody (BC α GP). Brevican full-length is detected at ≈ 145 kDa and a band at 80 kDa representing the C-terminal proteolytic fragment. Most brevican is found in the soluble fractions, especially in S2. However, brevican is also detected in membrane fractions and associated with synaptosomes (Syn) as described earlier (1). No signal at corresponding molecular weight was observed in brain lysates from brevican knock-out mice (H_{ko}). We evaluated the fraction of cleaved compared to full-length brevican by calculating the ratio of the intensity of the 80 kDa band divided by the full-length band in each sample. Interestingly we found that the ratio decreases in the membranous fractions, suggesting a high solubility and low membrane binding of the 80 kDa fragment (n = 3). B) Western blot probed with anti N-terminal (G1) domains (ms α brevican) also detected most brevican in the soluble fractions. No band is found in the brain homogenate from knockout mice. Interestingly the ratio of the 53 kDa N-terminal fragment to the full-length protein revealed a stronger attachment of the 53 kDa fragment to membranes compared to the full-length protein (n = 4). C) Western blot of brain fractions probed with neo-antibody. Highest signal was found in the soluble fraction. No band was detected at corresponding size in homogenate from knock-out mice. Quantification of the intensity of the 53 kDa band revealed that most of the ADAMTS4-derived fragment was in S2 (n=4). H = Homogenate; S1 = Supernatant 1 (1,000 x g), S2 = Supernatant 2 (14,000 x g); P2 = $\text{Pellet}_{14,000 \text{ x g}}$, Syn = Synaptosomes; My = Myelin; LM = Light membranes.

1. Seidenbecher CI, Richter K, Rauch U, Fassler R, Garner CC, Gundelfinger ED. Brevican, a chondroitin sulfate proteoglycan of rat brain, occurs as secreted and cell surface glycosylphosphatidylinositol-anchored isoforms. J Biol Chem. 1995;270(45):27206-12.

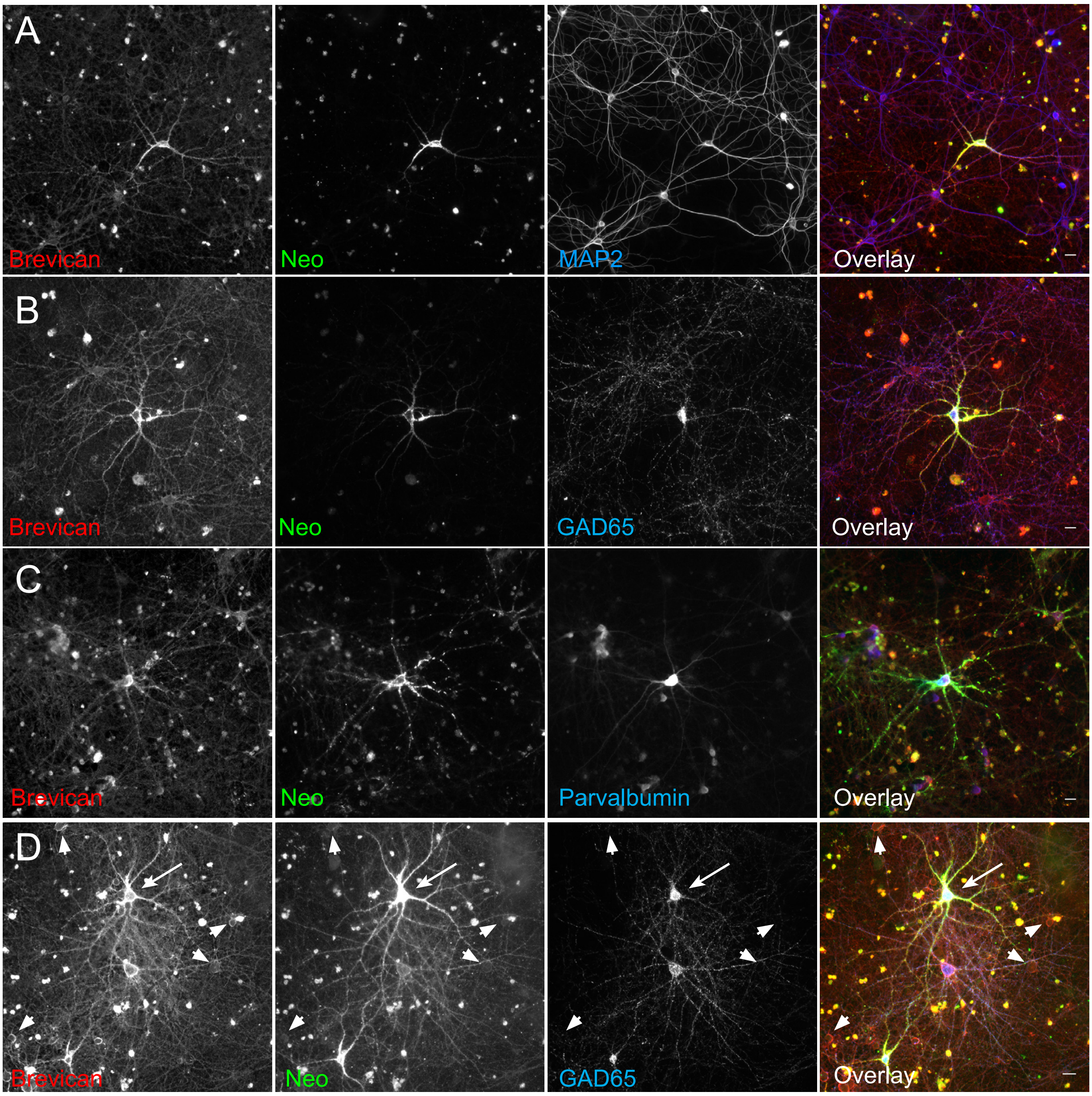

Supplementary Figure 2.

Brevican is found in diffuse ECM as well as in PNN-like structures in dissociated cortical neurons at DIV24. A) Brevican central domain antibody labels virtually all cells in the cultures but a subset of cells is more intensely labeled. Strongest neo staining is found in the same subset of cells, which were found to be GAD65- (B) and parvalbumin- (C) positive. D) Higher magnification shows GAD65 positive neurons with strong ECM labeling (arrow) and GAD65 negative ones with less pronounced, diffuse ECM (arrowheads) (scale bar = 10 μm).
